# Advanced respiratory mechanics assessment in mechanically ventilated obese and non-obese patients with or without acute respiratory distress syndrome

**DOI:** 10.1186/s13054-023-04623-2

**Published:** 2023-09-04

**Authors:** François M. Beloncle, Jean-Christophe Richard, Hamid Merdji, Christophe Desprez, Bertrand Pavlovsky, Elise Yvin, Lise Piquilloud, Pierre-Yves Olivier, Dara Chean, Antoine Studer, Antonin Courtais, Maëva Campfort, Hassene Rahmani, Arnaud Lesimple, Ferhat Meziani, Alain Mercat

**Affiliations:** 1 Medical ICU, University Hospital of Angers, Vent’Lab, University of Angers, 4 Rue Larrey, 49933 Angers Cedex 9, France; 2 CNRS, INSERM 1083, MITOVASC, University of Angers, Angers, France; 3 Med2Lab, ALMS, Antony, France; 4 Medical ICU, University Hospital of Strasbourg, University of Strasbourg, Strasbourg, France; 5 UMR 1260, Regenerative Nanomedicine (RNM), FMTS, INSERM (French National Institute of Health and Medical Research), Strasbourg, France; 6 Adult Intensive Care Unit, University Hospital and University of Lausanne, Lausanne, Switzerland

**Keywords:** Mechanical ventilation, Acute lung injury, Chest wall mechanic, Airway closure, Pleural pressure, Esophageal pressure

## Abstract

**Background:**

Respiratory mechanics is a key element to monitor mechanically ventilated patients and guide ventilator settings. Besides the usual basic assessments, some more complex explorations may allow to better characterize patients’ respiratory mechanics and individualize ventilation strategies. These advanced respiratory mechanics assessments including esophageal pressure measurements and complete airway closure detection may be particularly relevant in critically ill obese patients. This study aimed to comprehensively assess respiratory mechanics in obese and non-obese ICU patients with or without ARDS and evaluate the contribution of advanced respiratory mechanics assessments compared to basic assessments in these patients.

**Methods:**

All intubated patients admitted in two ICUs for any cause were prospectively included. Gas exchange and respiratory mechanics including esophageal pressure and end-expiratory lung volume (EELV) measurements and low-flow insufflation to detect complete airway closure were assessed in standardized conditions (tidal volume of 6 mL kg^−1^ predicted body weight (PBW), positive end-expiratory pressure (PEEP) of 5 cmH_2_O) within 24 h after intubation.

**Results:**

Among the 149 analyzed patients, 52 (34.9%) were obese and 90 (60.4%) had ARDS (65.4% and 57.8% of obese and non-obese patients, respectively, *p* = 0.385). A complete airway closure was found in 23.5% of the patients. It was more frequent in obese than in non-obese patients (40.4% vs 14.4%, *p* < 0.001) and in ARDS than in non-ARDS patients (30% vs. 13.6%, *p* = 0.029). Respiratory system and lung compliances and EELV/PBW were similarly decreased in obese patients without ARDS and obese or non-obese patients with ARDS. Chest wall compliance was not impacted by obesity or ARDS, but end-expiratory esophageal pressure was higher in obese than in non-obese patients. Chest wall contribution to respiratory system compliance differed widely between patients but was not predictable by their general characteristics.

**Conclusions:**

Most respiratory mechanics features are similar in obese non-ARDS and non-obese ARDS patients, but end-expiratory esophageal pressure is higher in obese patients. A complete airway closure can be found in around 25% of critically ill patients ventilated with a PEEP of 5 cmH_2_O. Advanced explorations may allow to better characterize individual respiratory mechanics and adjust ventilation strategies in some patients.

*Trial registration* NCT03420417 ClinicalTrials.gov (February 5, 2018).

**Supplementary Information:**

The online version contains supplementary material available at 10.1186/s13054-023-04623-2.

## Introduction

Respiratory mechanics is a key element in clinical practice to monitor mechanically ventilated patients and guide ventilator settings [1]. Respiratory system compliance (*C*_RS_) has been shown to correlate with the amount of aerated lung [2]. In addition, an increased respiratory system driving pressure (DP_RS_) has been shown to be associated with an increased risk of mortality in patients with ARDS [3, 4]. Beside this “basic” respiratory mechanics assessment, some more complex explorations, including in particular the evaluation of chest wall mechanics and the detection of complete airway closure, may allow to better characterize respiratory mechanics and personalize ventilator settings [5–7]. Instead of considering the respiratory system as a whole, partitioning it into the lung and the chest wall using esophageal pressure measurements has thus been proposed to estimate transpulmonary pressures and determine the amount of applied airway pressure, which is spent to inflate the lung and the one spent to displace the chest wall [8–10]. The airway closure, a phenomenon recently highlighted in ARDS patients, may also impact respiratory mechanics assessment and ventilatory management [5]. Complete airway closure leads to an absence of communication between proximal airways and alveoli when airway pressure is below the level of the so-called airway opening pressure (AOP). Practically, in case of complete airway closure, no gas enters the lung during insufflation until AOP has been overcome. Complete airway closure may thus lead to driving pressure overestimations and compliance underestimations when AOP is not considered in their calculations. In addition, a PEEP setting below the AOP level may promote inflammation due to cyclic airway closure and favor atelectasis [11]. Its detection at the bedside requires performing a low flow pressure–volume or pressure–time curve [5]. Some data suggest that this “advanced” respiratory mechanics assessment may be particularly relevant in obese patients [12–14]. Obesity is a major public health issue with a prevalence around 35% in adults in the United States of America and 13% worldwide and impacts critically ill patients’ management [15–17]. Most of the literature describing respiratory mechanics in obese patients comes however from the postsurgical setting and data concerning respiratory mechanics and in particular chest wall mechanics of critically ill obese patients remain scarce. Furthermore, the impact of obesity on respiratory mechanics has not been specifically assessed in patients fulfilling or not ARDS criteria. The main aim of this study was to comprehensively assess respiratory mechanics in obese and non-obese patients with or without ARDS and to study the additional value of an advanced respiratory mechanics evaluation (including esophageal pressure measurements and complete airway closure detection) in these patients compared to a basic evaluation (based on airway pressure monitoring). For this purpose, we prospectively measured gas exchange and respiratory mechanics in standardized conditions with the same positive end-expiratory pressure (PEEP) level and the same normalized tidal volume (Vt) in all intubated patients in two ICUs.

## Patients and methods

### Patients’ selection

All patients admitted from March 2018 to January 2020 in two academic hospital ICUs (Angers and Strasbourg, France) intubated and mechanically ventilated for any cause were prospectively included in the study within 24 h after intubation. Exclusion criteria were age < 18 years, pneumothorax, contraindication to esophageal pressure measurement, and use of extracorporeal membrane oxygenation (ECMO) at the time of inclusion. Patients admitted after a cardiac arrest were excluded from the analysis and reported in another publication [18]. Airway pressure and flow recordings of these patients were used to describe a novel approach to assess AOP [19].

### Study protocol

#### Settings

All patients received deep sedation and neuromuscular blockers at the time of measurements and were ventilated using an Engström^®^ or R860^®^ ventilator (GE Healthcare, Madison, WI, USA) in supine semi-recumbent position (head of the bed elevated at 30°). Respiratory mechanics and gas exchange were assessed under standardized conditions (volume-controlled ventilation with a Vt of 6 mL kg^−1^ of predicted body weight (PBW) and constant inspiratory flow of 60 L min^−1^ and PEEP of 5 cm H_2_O). Respiratory rate was adjusted by the attending physician (up to 35/min), and the fraction of inspired oxygen (FiO_2_) was set for pulsed oxygen saturation between 92 and 98%.

Esophageal pressure was measured with a specific nasogastric feeding tube equipped with an esophageal balloon (Nutrivent^®^ catheter, Sidam, San Giacomo Roncole, Italy) and connected to the auxiliary pressure transducer of the ventilator [9, 20]. The balloon was consecutively inflated to target a filling volume of 2, 3, and 4 mL. For the measurements, the filling volume was set as the lowest volume between 2 and 4 mL associated with the largest tidal swing of esophageal pressure during the insufflation of the Vt [21]. In addition, to avoid balloon overfilling, the balloon filling was stopped if a sudden and significant increase of the baseline esophageal pressure was observed. The correct position of the esophageal balloon was then checked by chest X-rays and an occlusion test [9, 20]. The ratio of esophageal pressure swing over airway pressure swing (Δ*P*_es_/Δ*P*_aw_) during the occlusion test was considered as acceptable if it was between 0.8 and 1.2.

To normalize volume history, a recruitment maneuver was performed in volume-controlled ventilation in absence of hemodynamic instability by increasing PEEP level up to 20 cmH_2_O for 1 min (maximum plateau pressure (*P*_Plat_) of 40 cmH_2_O). PEEP level was then switched back to 5 cmH_2_O.

Flow, airway pressure, and esophageal pressure–time curves were recorded using a dedicated computer connected to the ventilator with a 40 ms sampling time for offline analysis.

#### Measurements

All esophageal pressure signal recordings were independently inspected by two investigators blind to the other clinical data, and those considered non-valid were excluded from the analyses including esophageal pressure data.

Inspiratory and expiratory occlusion maneuvers were performed to measure *P*_Plat_, inspiratory esophageal pressure (*P*_eso inspi_), total PEEP, and expiratory esophageal pressure (*P*_eso expi_).

Abdominal pressure (*P*_abdo_) was measured using an intravesical catheter.

An arterial blood gas was performed after 15 min free of any occlusion maneuver, and EELV was measured at PEEP 5 cmH_2_O using the nitrogen washout-washin technique (E-COVX module sensor^®^, GE Healthcare) [22].

A low-flow inflation (5 L min^−1^, Vt = 8 mL kg^−1^ PBW) was then performed after a prolonged exhalation to PEEP 5 cmH_2_O.

Complete airway closure and corresponding AOP were identified by the inspection of the pressure–volume curves as previously described [5, 23].

#### Calculated variables

DP_RS_ was computed as the difference between *P*_Plat_ and total PEEP. *C*_RS_ was computed as the expired tidal volume (Vte) divided by DP_RS_. The respiratory system elastance (*E*_RS_) was equal to 1/*C*_RS_. The respiratory system resistance was computed as the difference between peak airway pressure and *P*_Plat_ divided by the inspiratory flow.

The difference between P1 and *P*_Plat_ (ΔP1–*P*_Plat_) with P1 defined as airway pressure at first zero flow was computed to assess viscoelastic properties of the lung and chest wall tissues and pendelluft phenomenon [24].

Inspiratory (*P*_Linspi_) and expiratory transpulmonary pressures (*P*_Lexpi_) were computed as the difference between *P*_Plat_ and *P*_eso inspi_, and between total PEEP and *P*_eso expi_, respectively.

The lung driving pressure (DP_L_) was computed as the difference between *P*_Linspi_ and *P*_Lexpi_ [25]. The lung compliance (*C*_L_) was computed as Vte divided by DP_L_. The lung elastance (*E*_L_) was equal to 1/*C*_L_. The chest wall compliance (*C*_CW_) was computed as Vte divided by the difference between *P*_eso inspi_ and *P*_eso expi_.

The elastance ratio (*E*_L_/*E*_RS_) was calculated to assess the respective effects of the airway pressure on the lung and the chest wall [8, 10]. Plateau pressure of the lung (*P*_Plat Lung_) was computed as *P*_Plat_ multiplied by *E*_L_/*E*_RS_ [26].

As *P*_Plat Lung_ and *P*_Lexpi_ can be considered respectively as good surrogates of the inspiratory transpulmonary pressure in the nondependent lung and the expiratory transpulmonary pressure in the dependent lung [27], we computed the lung stress as the difference between *P*_Plat lung_ and *P*_Lexpi_, to assess the real stress applied to the lung across the ventral to dorsal axis.

DP_RS-AOP_ and *C*_RS-AOP_ were computed as DP_RS_ and *C*_RS_ using AOP instead of total PEEP in the calculations in patients with an AOP higher than total PEEP.

The ratios *C*_RS_/PBW, *C*_L_/PBW, and EELV/PBW were calculated to normalize *C*_RS_, *C*_L,_ and EELV to the gender and the height of the patient. PBW was calculated using the previously published formula [28].

Dead space was assessed using ventilatory ratio, which was computed as minute ventilation (mL/min) × PaCO_2_ (mmHg)] / (PBW (kg) × 100 × 37.5) [29, 30].

#### Other collected data

Age, height, weight, past medical history of chronic respiratory disease, immunodepression, Sequential Organ Failure Assessment (SOFA) score [31], and Simplified Acute Physiologic Score II (SAPS II) [32] were collected on the day of admission.

The lung opacities were independently assessed on chest X-rays by two experienced investigators blind to clinical data.

The diagnosis of ARDS was performed using the criteria of the Berlin definition by an adjudication committee blind to respiratory mechanics data [33].

Survival was assessed at day 60 after inclusion.

The number of ventilator-free days at day 28 was defined as the number of days between day 1 and day 28 on which patients breathed without assistance. A value of 0 ventilator-free day was assigned for patients who died before day 28.

### Statistical analysis

Results are presented as median [interquartile range] and number (percentage). Normality of the variables was assessed using the D’Agostino & Pearson test. The study population was divided into four groups according to the presence of obesity or not (BMI < or ≥ 30 kg m^−2^) and the presence of ARDS or not at the time of respiratory mechanics assessment [33]. All the patients with ARDS were also compared to those without ARDS, and all obese patients were compared to non-obese patients. In addition, the population was divided into three groups according to whether *E*_L_/*E*_RS_ ratio was lower than the first quartile of *E*_L_/*E*_RS_ of the study population (*Low E*_L_*/E*_RS_), higher than the third quartile (*high E*_L_*/E*_RS_), or in-between *(medium E*_L_*/E*_RS_). We planned to enroll more than 140 patients to be able to detect 10% absolute changes between the defined patients’ groups in the main physiological variables assessed in the study (with 80% power at a two-sided type I error of 0.05). The groups were compared using Kruskal–Wallis test, Mann–Whitney *U*-test, *t*-test, Fischer or Chi^2^ test, as appropriate according to data distribution. Bonferroni correction was applied for multiple pairwise comparisons. Correlations were analyzed using Spearman test. All tests were performed with a type I error set at 0.05. The statistical analysis was performed using R version 3.6.2 (R Core Team 2019, Vienna, Austria, https://www.R-project.org/).

## Results

### Main patients’ characteristics

One hundred and sixty-four patients were included in the study. One hundred and forty-nine of them were included in the analysis (15 patients were excluded because of lack of data due to technical issues in recordings or major deviations in study protocol). Valid esophageal pressure measurements were analyzed in 124 patients. Respiratory mechanics and gas exchange were assessed 10 [3.5–22] hours after intubation.

Fifty-two patients (34.9%) were obese. Ninety (60.4%) patients fulfilled ARDS criteria (65.4% of obese and 57.8% of non-obese patients, *p* = 0.385). The main patient’s characteristics categorized according to the presence or not of obesity and/or ARDS are presented in Table 1 and Additional file 1: Table S1.

**Table 1:** Main characteristics of the patients

	All patients *n* = 149	Non-obese non-ARDS *n* = 41	Non-obese ARDS *n* = 56	Obese non-ARDS *n* = 18	Obese ARDS *n* = 34	Overall comparison *p*-value
Age—years	65 [56–77]	69 [56–80]	64 [53–74]	66 [62–75]	64 [59–76]	0.65
Male sex—*n*	91 (61.1)	25 (61.0)	32 (57.1)	12 (66.7)	22 (64.7)	0.85
Height—cm	168 [160–174]	168 [159–174]	166 [160–175]	167 [161–174]	170 [163–174]	0.65
BMI—kg m^−2^	26 [23–31]	24 [21–24]	24 [22–25]	34 [30–36]***^, ###^	34 [31–37]***^, ###^	< 0.001
Abdominal perimeter—cm	104 [93–115]	93 [83–98]	98 [93–107]	116 [110–119]**^, ##^	120 [115–134]**^, ###^	< 0.001
SOFA at enrollment	8 [6–11]	7 [6–9]	10 [6–12]	8 [6–9]	8 [5–10]	0.087
Non-pulmonary SOFA at enrollment	5 [3.5–8]	5 [4–7]	6 [4–8]	5 [4–6]	4 [2–7]	0.35
SAPS II at enrollment	50 [39–63]	50 [40–63]	52 [40–66]	50 [38–70]	46 [35–57]	0.55
Chronic pulmonary disease—n	36 (24.2)	8 (19.5)	7 (12.5)	6 (33.3)	15 (44.1) ^#^	0.012
Immunodepression—n	18 (12.1)	5 (12.2)	10 (17.9)	1 (5.6)	2 (5.9)	0.294
*Cause of ICU admission—n*						
Pneumonia	57 (38.3)	3 (7.3)	37 (66.1) ***	0 (0) ^###^	17 (50) ***^,§§^	< 0.001
Hydrostatic pulmonary edema	14 (9.4)	6 (14.6)	0 (0)*	8 (44.4) ^###^	0 (0) ^§§§^	< 0.001
Non-pulmonary Sepsis	21 (14.1)	6 (14.6)	7 (12.5)	0 (0)	8 (14.8)	0.134
Acute exacerbation of obstructive pulmonary disease	3 (2)	3 (7.3)	0 (0)	0 (0)	0 (0)	0.045
Neurologic conditions/coma	28 (18.8)	17 (41.5)	3 (5.4)***	6 (33.3) ^##^	2 (3.7)**	< 0.001
Metabolic disorder	3 (2)	0 (0)	1 (1.8)	2 (11.1)	0 (0)	0.028
Other	23 (15.4)	6 (14.6)	8 (14.3)	2 (11.1)	7 (13)	0.795
Survival at day 60—n	101 (67.8)	33 (80.5)	31 (55.4)	13 (72.2)	24 (70.6)	0.064
Number of ventilator-free days at day 28—days	13 [0–23]	23 [0–26]	1 [0–18]***	19 [2–25]^#^	12 [0–19]*	< 0.001

Data are presented as median [interquartile range] or number (percentage)

ARDS, Acute Respiratory Distress Syndrome; BMI, Body Mass Index; SOFA, Sequential Organ Failure Assessment; SAPS II, Simplified Acute Physiology Score II

*p < 0.05, **p < 0.01, ***p < 0.001 compared to non-obese non-ARDS patients; ^#^*p* < 0.05, ^##^*p* < 0.01, ^###^*p* < 0.001 compared to non-obese ARDS patients; ^§§^*p* < 0.01, ^§§§^*p* < 0.001 compared to obese non-ARDS patients

### Gas exchange

Gas exchange in the patients categorized according to the presence or not of obesity and/or ARDS is presented in Table 2 and Additional file 1: Table S2. PaO_2_/FiO_2_ ratio was not different between obese non-ARDS, obese ARDS, and non-obese ARDS patients but was lower in non-obese non-ARDS patients than in the other groups of patients. It tended to be lower in obese than in non-obese patients and was lower in ARDS than in non-ARDS patients. Ventilatory ratio was not different between obese non-ARDS, obese ARDS, and non-obese ARDS patients but was lower in non-obese non-ARDS patients than in non-obese ARDS and obese ARDS patients. It was higher in obese than in non-obese patients and in ARDS than in non-ARDS patients. PaO_2_/FiO_2_ ratio and ventilatory ratio correlated with BMI in non-ARDS patients but not in ARDS patients (Additional file 1: Fig. S1A and B).

**Table 2:** Gas exchange and respiratory mechanics

	All patients *n* = 149	Non-obese Non-ARDS *n* = 41	Non-obese ARDS *n* = 56	Obese non-ARDS *n* = 18	Obese ARDS *n* = 34	Overall comparison *p*-value
RR—min^−1^	25 [20–29]	20 [19–25]	26 [23–29]***	24 [20–27]	30 [24–29]***	< 0.001
VE—L min^−1^	9.6 [7.7–11.3]	8.4 [6.8–10.2]	9.9 [8–11.0]*	9.2 [8.2–9.9]	10.8 [9.5–11.9]**	0.002
FiO_2_—%	50 [30–70]	30 [24–37]	60 [40–80]***	60 [40–60]**	52 [40–70]***	< 0.001
PaO_2_—mmHg	79 [69–96]	88 [69–129]	74 [63–87]*	74 [71–94]	82 [70–91]	0.038
PaO_2_/FiO_2_—mmHg	183 [120–255]	314 [243–378]	129 [100–188]***	174 [121–242]***	157 [121–208]***	< 0.001
PaCO_2_—mmHg	40 [35–47]	36 [32–37]	41 [35–50]*	40 [37–48]	42 [39–46]**	0.009
Ventilatory ratio	1.3 [1.6–2]	1.3 [1.1–1.5]	1.8 [1.4–2.3]***	1.8 [1.1–2.2]	1.9 [1.6–2.1]***	< 0.001
Patients with complete airway closure > 5 cmH_2_O—n	35 (23.5)	4 (9.8)	10 (17.9)	4 (22.2)	17 (50.0) **^, #^	< 0.001
AOP in patients with complete airway closure > 5 cmH_2_O—cmH_2_O	8.5 [7.5–11]	9.5 [6.5–12.5]	9 [7.5–11]	8.5 [7–10]	8.5 [8–10]	0.84
PEEP_tot_—cmH_2_O	6 [5.5–7.5]	5.5 [5–6]	6 [5.5–7.5]	6.5 [5.5–6.5]	7.5 [6–9.5]***, ^##, $$^	< 0.001
*P*_Plat_—cmH_2_O	15 [13–18]	12.5 [11–14.5]	15.5 [13.5–18.5]***	15.5 [14–16.5]*	17 [15–20.5]***	< 0.001
*C*_RS_—mL cmH_2_O^−1^	44 [36–56]	55 [45–73]	41 [31–51]**	41 [36–51]*	43 [35–53]**	< 0.001
*C*_RS-AOP_—mL cmH_2_O^−1^	46 [36–57]	56 [45–73]	41 [31–51]**	41 [36–52]*	43 [36–54]*	< 0.001
DP_RS_—cmH_2_O	8.5 [7–11]	6.5 [5.5–8.5]	9 [6.5–11.5]***	9.5 [8.5–10]**	9 [7.5–11]**	< 0.001
DP_RS-AOP_—cmH_2_O	8.5 [6.5–10]	6.5 [5.5–8.5]	9.0 [6.5–11.5]**	9 [7.5–10.0]**	8.5 [7.5–10.5]**	< 0.001
R_RS_—cmH_2_O L^−1^ s^−1^	17 [14–21]	16 [14.5–21]	16.5 [13.5–20]	17 [14–18.5]	18 [14.5–22.5]	0.34
ΔP1–*P*_Plat_—cmH_2_O	2.1 [1.1–3.4]	1.3 [0.9–2.7]	2.4 [1.3–4.2]	2.3 [1.7–2.9]	2.8 [1.1–5.2]	0.128
DP_L_—cmH_2_O	5 [4–7.5]	4 [3–6]	5.5 [4–8.5]*	6.5 [5.5–8]*	5.5 [4.5–7.5]*	0.005
*P*_Plat Lung_—cmH_2_O	9.5 [7.5–12.5]	7.5 [6–9.5]	10 [8–13]*	11 [9.5–13]*	11 [9–12.5]**	0.002
Lung Stress—cmH_2_O	10 [7.5–14.5]	8 [4–10.5]	10 [8–14]*	14.5 [13.5–16.5]**	12.5 [9–16]*	0.002

Data are presented as median [interquartile range] or number (percentage)

RR, Respiratory Rate; VE, Minute ventilation; FiO_2_, Fraction of inspired oxygen; PaO_2_, Partial pressure of arterial oxygen; PaCO_2_, Partial pressure of arterial carbon dioxide; AOP, Airway Opening Pressure; PEEP_tot_, Total Positive End-Expiratory Pressure; *P*_Plat_, Plateau Pressure; *C*_RS,_ Respiratory System Compliance; *C*_RS-AOP_, *C*_RS_ using AOP instead of PEEP_tot_ in the calculation; DP_RS,_ Respiratory System Driving Pressure; DP_RS-AOP_, DP_RS_ using AOP instead of PEEP_tot_ in the calculation; R_RS_, Respiratory System Resistance; ΔP1–*P*_Plat_, Difference between P1 and *P*_Plat_ with P1 defined as airway pressure at first zero flow; DP_L_, Lung Driving Pressure; *P*_Plat Lung_, Plateau Pressure of the Lung. Lung stress was defined as the difference between *P*_Plat Lung_ and the expiratory transpulmonary pressure

*p < 0.05, **p < 0.01, ***p < 0.001 compared to non-obese non-ARDS patients, ^#^*p* < 0.05, ^##^*p* < 0.01, ^###^*p* < 0.001 compared to non-obese ARDS patients; ^$$^*p* < 0.01 compared to obese non-ARDS patients

### Airway closure and driving pressure

A complete airway closure assessed with a PEEP of 5 cmH_2_O was found in 23.5% of the patients. It was found in some patients of the four groups but was more frequent in obese ARDS patients than in non-obese non-ARDS and non-obese ARDS patients (Table 2). It was more frequent in obese than in non-obese patients (40.4% vs. 14.4%, *p* < 0.001) and in ARDS than in non-ARDS patients (30% vs. 13.6%, *p* = 0.029) (Additional file 1: Table S2).

DP_RS_ and DP_RS-AOP_ in the patients categorized according to the presence or not of obesity and/or ARDS are presented in Table 2 and Additional file 1: Table S2. Considering the whole population, DP_RS-AOP_ was different from DP_RS_ in 15 (10.1%) patients. In these 15 patients, the difference between DP_RS_ and DP_RS-AOP_ was 1.5 [1–3] cmH_2_O.

ΔP1-*P*_Plat_ was not different between obese and non-obese patients but was higher in patients with ARDS than in those who did not meet ARDS criteria (Table 2 and Additional file 1: Table S2).

### Lung volumes and compliances

**Fig. 1:**
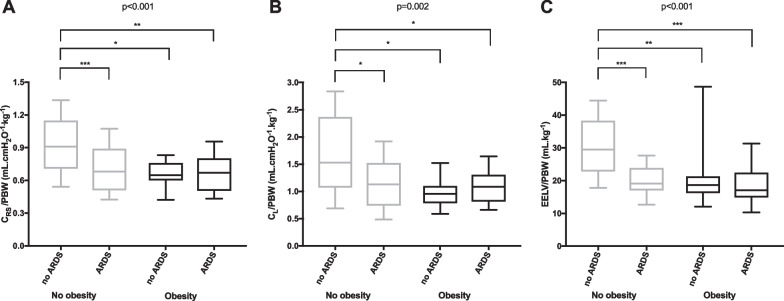
Distribution of respiratory system compliance (*C*_RS_/PBW, **A**), lung compliance (*C*_L_/PBW, **B**), and end-expiratory lung volume (EELV/PBW, **C**) normalized to predicted body weight in patients categorized according to the presence or not of obesity and acute respiratory distress syndrome (ARDS). Boxplots display medians, 10th, 25th, 75th, and 90th percentiles. *p*-values represent the overall comparisons between the four groups of patients. *, *p* < 0.05; **, *p* < 0.01; ***, *p* < 0.001 (pairwise comparisons with Bonferroni correction)

*C*_RS_/PBW, *C*_L_/PBW, and EELV/PBW were not different between obese non-ARDS patients and obese or non-obese ARDS patients but were higher in non-obese non-ARDS patients than in the other groups of patients (Fig. 1). Those parameters were lower in obese than in non-obese patients and in ARDS than in non-ARDS patients (Additional file 1: Fig. S2). Similar results were found when considering the AOP in the calculation of respiratory system compliance (*C*_RS-AOP_/PBW, Additional file 1: Fig. S3). The correlations between *C*_RS_/PBW and BMI and between EELV/PBW and BMI in ARDS and non-ARDS patients are presented in additional data (Additional file 1: Fig. S4A and B).

*C*_RS_ correlated well with EELV in obese and non-obese patients with or without ARDS (Additional file 1: Fig. S5).

### Chest wall mechanics

**Fig. 2:**
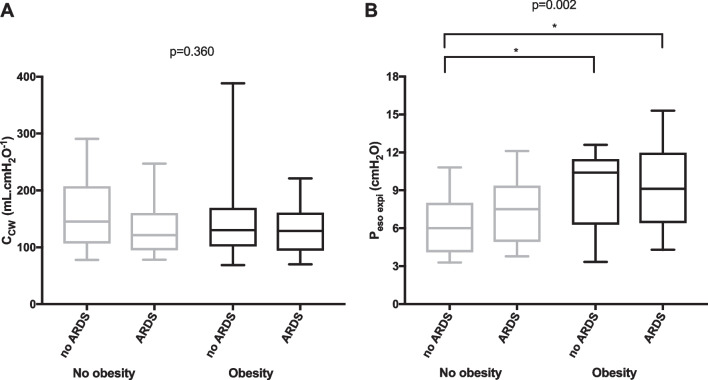
Distribution of chest wall compliance (C_CW_, **A**) and end-expiratory esophageal pressure (*P*_eso expi_, **B**) in patients categorized according to the presence or not of obesity and acute respiratory distress syndrome (ARDS). Boxplots display medians, 10^th^, 25^th^, 75^th^, and 90^th^ percentiles. *p*-values represent the overall comparisons between the four groups of patients. *, *p* < 0.05 (pairwise comparisons with Bonferroni correction)

**Fig. 3:**
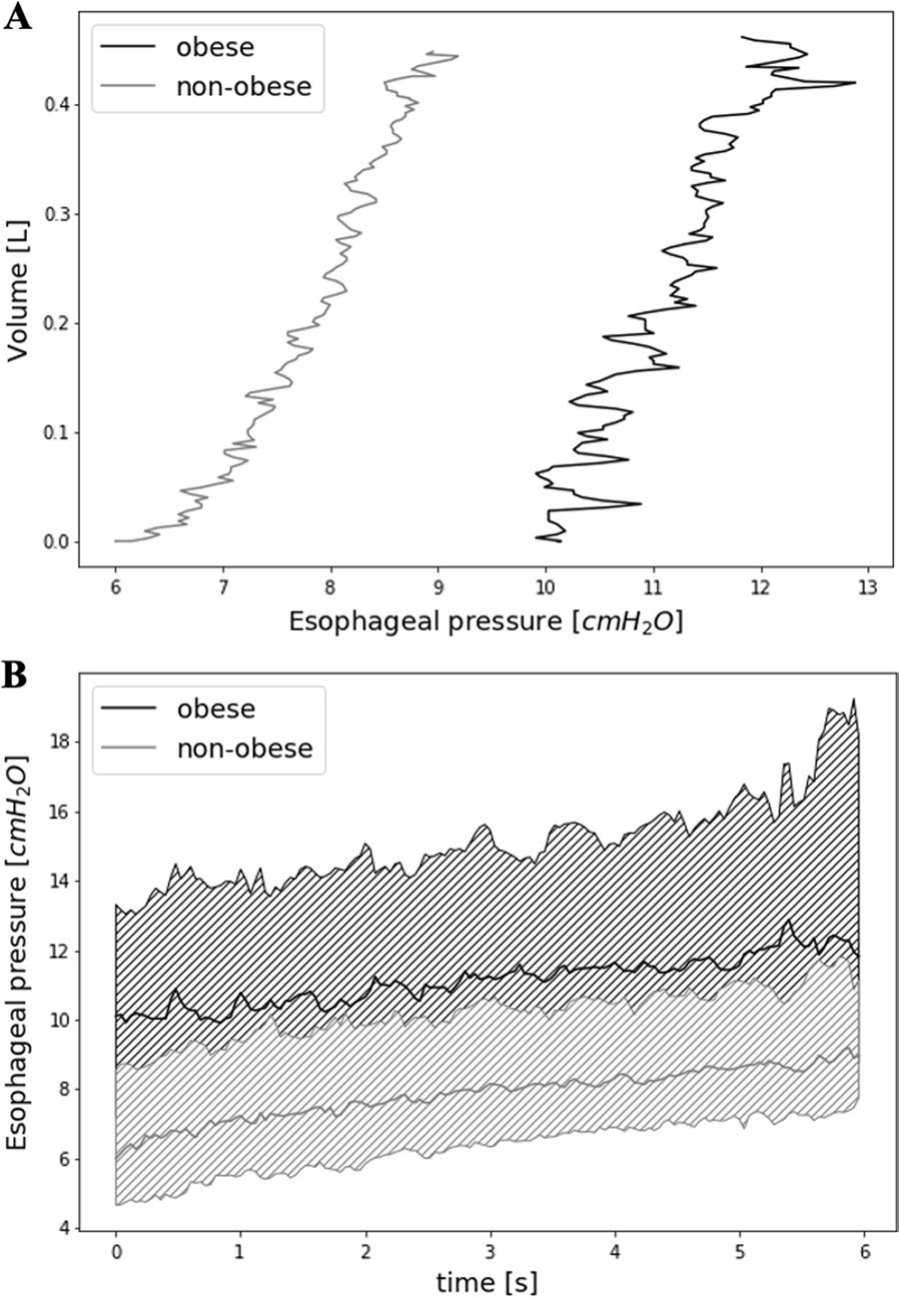
Esophageal pressure–volume (**A**) and esophageal pressure–time (**B**) curves during low-flow insufflation (5L min^−1^) in obese and non-obese patients. **A** Black and gray lines represent median values of all obese and non-obese patients included in the study, respectively. **B** Black and gray lines represent median values and interquartile range of all obese and non-obese patients included in the study, respectively

C_CW_ was not different between the four groups of patients and between obese and non-obese patients or between ARDS and non-ARDS patients but *P*_eso expi_ was higher in obese patients with or without ARDS than in non-obese non-ARDS patients (Fig. 2 and Additional file 1: Fig. S6). *P*_eso expi_ was higher in obese patients than in non-obese patients but was not significantly different between ARDS and non-ARDS patients. BMI was not correlated with C_CW_ but was correlated with *P*_eso expi_ in patients with or without ARDS (Additional file 1: Fig. S7A and B). Esophageal pressure–volume and esophageal pressure–time curves during low-flow insufflation of obese and non-obese patients are presented in Fig. 3.

*P*_eso expi_ and C_CW_ were not correlated with *P*_abdo_ (*p* = 0.331, rho = 0.181 and *p* = 0.183, rho = − 0.242, respectively; *n* = 32). *P*_eso expi_ was correlated with EELV/PBW (*p* < 0.001, rho = − 0.365) and *C*_RS_/PBW (*p* = 0.010, rho = − 0.231) but not with AOP (*p* = 0.306, rho = 0.193).

The *E*_L_/*E*_RS_ ratio in the whole population was 0.64 [0.57–0.72]. It was not different between the four groups of patients and between those with and without obesity or between those with and without ARDS (Additional file 1: Fig. S8). The main characteristics of the patients with low *E*_L_/*E*_RS_ (i.e., in whom chest wall compliance markedly impacts respiratory system compliance) were not different from those with medium or high *E*_L_/*E*_RS_ (Additional file 1: Table S3). But patients with low *E*_L_/*E*_RS_ had higher *C*_RS_, *C*_L,_ and EELV/PBW and were less hypoxemic than patients with higher *E*_L_/*E*_RS_ (Additional file 1: Table S4).

### Lung driving pressure, plateau pressure of the lung and lung stress

DP_L,_ *P*_Plat lung_, and lung stress were not different between obese non-ARDS patients and obese or non-obese ARDS patients but were lower in non-obese non-ARDS patients than in the other groups of patients (Table 2 and Additional file 1: Table S2).

## Discussion

The main results of the study can be summarized as follows:

1. Oxygenation, *C*_RS_, *C*_L_, and EELV were similarly altered in obese patients without ARDS and patients with ARDS (either obese or non-obese).
2. *P*_eso expi_ was higher in obese patients than in non-obese patients but C_CW_ did not differ between these groups of patients. Chest wall contribution to *C*_RS_ expressed by the *E*_L_/*E*_RS_ ratio was widely distributed and was not predictable by patient’s general characteristics.
3. Complete airway closure was observed in all groups of patients but was more frequently found in obese than in non-obese patients and in ARDS than in non-ARDS patients. Ignoring airway closure led to an overestimation of DP_RS_ in almost 17% of obese patients.

In the present series, gas exchange, *C*_RS_, *C*_L_, and EELV were similarly affected by obesity and ARDS in comparison with non-obese non-ARDS patients. These original findings can be explained by the reduction in lung volumes reported in both obese and ARDS patients. Chest wall mechanics differ however between obese and ARDS patients with higher *P*_eso expi_ in obese patients despite similar C_CW_. This increased *P*_eso expi_ may be related to the decreased EELV and the increased frequency of complete airway closure in these patients.

The impairments in lung volumes and chest wall mechanics that we measured in critically ill obese ARDS and non-ARDS patients are consistent with the observations previously reported by Coudroy et al*.* in a post hoc pooled analysis of two small cohorts of patients with ARDS [13]. In this work, *P*_eso expi_, but not C_CW_, was shown to correlate with BMI. Airway closure was also found to be more frequently observed in patients with higher BMI [13]. Based on CT scan analyses, Chiumello et al*.* reported lower lung gas volume and higher total superimposed pressure in obese ARDS compared to non-obese ARDS patients [34]. Noticeably, in this series, C_CW_ was similar in obese and non-obese ARDS patients, which is consistent with our observations but P_L expi_ did not differ.

In addition, our observations in critically ill patients are consistent with those reported in obese surgical patients [35–37]. Pelosi et al*.* found however a lower C_CW_ in morbidly obese patients [36]. This discrepancy with our results may be related to the higher BMI, and the strict supine position in which measurements were performed in Pelosi et al*.* study [36].

Our study is the first to systematically assess, soon after intubation, and according to a well-standardized protocol, the complete respiratory mechanics in a large series of non-selected patients including ARDS and non-ARDS patients. This methodological strength is of particular relevance to appreciate properly the roles played by obesity and ARDS since respiratory mechanics have been shown to significantly change over time under mechanical ventilation due to several confounding factors as the progressive increase in lung weight.

Our findings have important clinical implications especially since obesity is frequent in ICU patients [15]. The respiratory mechanics features observed in both obese and ARDS patients suggest that lung protective ventilation strategy could overall be similarly managed in these patients but no interventional study has so far specifically evaluated the potential benefit of such a strategy in obese non-ARDS patients [14]. In addition, our data suggest that advanced explorations may be of particular value to better assess respiratory mechanics and individualize ventilator settings. Interestingly, whereas basic respiratory mechanics assessments showed similar alterations in obese non-ARDS and non-obese ARDS patients, advanced explorations revealed that mechanisms involved were different in these two groups of patients. Differences in *P*_eso expi_ may thus lead to different PEEP settings when a positive *P*_L expi_ is considered as a goal to optimize ventilation [38, 39]. Moreover, our study shows that the *E*_L_/*E*_RS_ ratio may significantly differ between patients and cannot be easily predicted by the main patients’ characteristics. Esophageal pressure monitoring is thus needed to assess the contribution of C_CW_ to *C*_RS_. Furthermore, an assessment of airway closure may be systematically considered as this phenomenon impacts driving pressure measurements in around 10% of the patients (and even 17% of obese patients). Such alterations in obese patients respiratory mechanics may contribute to explain the absence of association between DP_RS_ and mortality observed in obese ARDS patients contrary to what was observed in non-obese ARDS patients [40]. In addition, a PEEP level set below the AOP may be associated with a higher risk of ventilator induced lung injury because of the heterogeneity of tidal ventilation distribution and atelectrauma [11].

Our study has several limitations. First, gas exchange and respiratory mechanics were assessed at only one PEEP level and lung recruitability was not directly evaluated. Higher PEEP could have been associated with different observations, but our study design allowed to assess all the patients in similar standardized and safe conditions. Second, we did not deduct the estimated pressure generated by the esophagus wall from the directly measured esophageal pressure [21]. However, our calibration procedure allowed to adjust the balloon filling volume to limit the risk of balloon overstretching, and the amplitude of the difference between the directly measured non-corrected esophageal pressure and the corrected value using the strategy proposed by Mojoli et al. is likely to be very small in this setting. Third, obesity may appear as a heterogeneous disease and some features may be observed only in morbidly obese (BMI > 40 kg m^−2^) or may vary according to the distribution of fat tissue. ARDS may also be considered as a heterogenous syndrome, and we did not distinguish ARDS caused by pulmonary and non-pulmonary disease. Last, ARDS Berlin definition may be difficult to apply in obese patients who are often hypoxemic and for whom condensations may be difficult to assess on chest X-rays. This difficult classification may contribute to explain why some authors found that obesity was associated with a higher risk of ARDS development [41]. Interestingly, in our study in which chest X-rays were independently assessed by two experienced investigators, and ARDS diagnosis was defined by an adjudication committee, ARDS was not found to be more frequent in obese than in non-obese patients.

## Conclusion

Basic respiratory mechanics and gas exchange features of obese patients are similar to those observed in non-obese ARDS patients. But an advanced assessment of respiratory mechanics allows to show that end-expiratory esophageal pressure, although largely distributed, is higher in obese patients. Chest wall compliance is not altered in obese or ARDS patients and is not easily predictable by patients’ general characteristics. A complete airway closure can be found in around 25% of critically ill patients ventilated with a PEEP of 5 cmH_2_O. Although it is more frequent in obese or ARDS patients, it can be observed in around 10% of non-obese non-ARDS patients. Advanced explorations including esophageal pressure and airway closure assessment can allow to better characterize individual respiratory mechanics and adjust ventilation strategies in some patients.

### Supplementary Information

**Additional file 1: Table S1.** Main characteristics of the patients according to the presence or not of ARDS and obesity. **Table S2.** Gas exchange and respiratory mechanics of the patients according to the presence or not of ARDS and obesity. **Table S3.** Main characteristics of the patients according to the ratio of lung to respiratory system elastance (*E*_L_/*E*_RS_). **Table S4.** Gas exchange and respiratory mechanics of the patients according to the ratio of lung to respiratory system elastance (*E*_L_/*E*_RS_). **Fig. S1.** Correlations between the ratio of partial pressure of arterial oxygen over fraction of inspired oxygen (PaO_2_/FiO_2_, **A**) and ventilatory ratio (VR, **B**), and body mass index (BMI) in patients with or without acute respiratory distress syndrome (ARDS). **Fig. S2.** Distribution of respiratory system compliance (*C*_RS_/PBW, **A**), lung compliances (*C*_L_/PBW, **B**) and end-expiratory lung volume (EELV/PBW, **C**) normalized to predicted body weight in patients categorized according to the presence or not of obesity and the presence or not of acute respiratory distress syndrome (ARDS). Boxplots display medians, 10th, 25th, 75th, and 90th percentiles. *p*-values represent the comparisons between obese and non-obese patients and between ARDS and non-ARDS patients. **Fig. S3.** Distribution of respiratory system compliances considering airway opening pressure normalized to predicted body weight (*C*_RS-AOP_/PBW). A. Patients categorized according to the presence or not of obesity and acute respiratory distress syndrome (ARDS). *p*-value represents the overall comparison between the four groups of patients. *, *p *< 0.05; 0**, *p *< 0.01; ***, *p *< 0.001 (pairwise comparisons with Bonferroni correction). **B** Patients categorized according to the presence or not of obesity and the presence or not of ARDS. *p*-values represent the comparisons between obese and non-obese patients and between ARDS and non-ARDS patients. Boxplots display medians, 10th, 25th, 75th, and 90th percentiles. **Fig. S4.** Correlations between respiratory system compliance (*C*_RS_/PBW, **A**) and end-expiratory lung volume normalized to predicted body weight (EELV/PBW, **B**) and body mass index (BMI) in patients with or without acute respiratory distress syndrome (ARDS). **Fig. S5.** Correlations between respiratory system compliance (*C*_RS_) and end-expiratory lung volume (EELV) at positive end-expiratory pressure of 5 cmH_2_O in obese and non-obese patients with or without acute respiratory distress syndrome (ARDS). **Fig. S6.** Distribution of chest wall compliance (CCW, **A**) and end-expiratory esophageal pressure (*P*_eso expi_, **B**) in patients categorized according to the presence or not of obesity and the presence or not of acute respiratory distress syndrome (ARDS). Boxplots display medians, 10th, 25th, 75th, and 90th percentiles. *p*-values represent the comparisons between obese and non-obese patients and between ARDS and non-ARDS patients. **Fig. S7.** Correlations between chest wall compliance (*C*_cw_, **A**) and expiratory esophageal pressure (*P*_eso expi_, **B**) and body mass index (BMI) in patients with or without acute respiratory distress syndrome (ARDS). **Fig. S8.** Distribution of lung to respiratory system elastance ratio (*E*_L_/*E*_RS_). **A** Patients categorized according to the presence or not of obesity and acute respiratory distress syndrome (ARDS). *p*-value represents the overall comparison between the four groups of patients. **B** Patients categorized according to the presence or not of obesity and the presence or not of ARDS. *p*-values represent the comparisons between obese and non-obese patients and between ARDS and non-ARDS patients. Boxplots display medians, 10th, 25th, 75th, and 90th percentiles.

## Data Availability

The datasets analyzed during the current study are available from the corresponding author on reasonable request.

## Abbreviations

AOP: Airway opening pressure
ARDS: Acute respiratory distress syndrome
*C*_CW_: Chest wall compliance
*C*_L_: Lung compliance
*C*_RS_: Respiratory system compliance
*C*_RS-AOP_: Respiratory system compliance using airway opening pressure instead of total positive end-expiratory pressure in the calculation in case of complete airway closure
DP_L_: Lung driving pressure
DP_RS_: Respiratory system driving pressure
DP_RS-AOP_: Respiratory system driving pressure using airway opening pressure instead of total positive end-expiratory pressure in the calculation in case of complete airway closure
EELV: End-expiratory lung volume
*E*_L_: Lung elastance
*E*_RS_: Respiratory system elastance
FiO_2_: Fraction of inspired oxygen
*P*_abdo_: Abdominal pressure
PaCO_2_: Partial pressure of arterial carbon dioxide
PaO_2_: Partial pressure of arterial oxygen
PBW: Predicted body weight
PEEP: Positive end-expiratory pressure
*P*_eso expi_: Expiratory esophageal pressure
*P*_eso inspi_: Inspiratory esophageal pressure
*P*_Linspi_: Inspiratory transpulmonary pressure
*P*_Lexpi_: Expiratory transpulmonary pressure
*P*_Plat_: Plateau pressure
*P*_Plat Lung_: Plateau pressure of the lung
ΔP1–*P*_Plat_: Difference between P1 and PPlat with P1 defined as airway pressure at first zero flow
SAPS II: Simplified acute physiologic score II
SOFA: Sequential organ failure assessment
Vt: Tidal volume
