# Supplementary material for: Advanced respiratory mechanics assessment in mechanically ventilated obese and non-obese patients with or without acute respiratory distress syndrome
Source: Crit Care. 2023 Sep 4;27:343. doi: 10.1186/s13054-023-04623-2 (PMC10476380; doi:10.1186/s13054-023-04623-2)
Supplement: Supplementary file 1 — Additional file 1: Table S1. Main characteristics of the patients according to the presence or not of ARDS and obesity. Table S2. Gas exchange and respiratory mechanics of the patients according to the presence or not of ARDS and obesity. Table S3. Main characteristics of the patients according to the ratio of lung to respiratory system elastance (EL/ERS). Table S4. Gas exchange and respiratory mechanics of the patients according to the ratio of lung to respiratory system elastance (EL/ERS). Fig. S1. Correlations between the ratio of partial pressure of arterial oxygen over fraction of inspired oxygen (PaO2/FiO2, A) and ventilatory ratio (VR, B), and body mass index (BMI) in patients with or without acute respiratory distress syndrome (ARDS). Fig. S2. Distribution of respiratory system compliance (CRS/PBW, A), lung compliances (CL/PBW, B) and end-expiratory lung volume (EELV/PBW, C) normalized to predicted body weight in patients categorized according to the presence or not of obesity and the presence or not of acute respiratory distress syndrome (ARDS). Boxplots display medians, 10th, 25th, 75th, and 90th percentiles. p-values represent the comparisons between obese and non-obese patients and between ARDS and non-ARDS patients. Fig. S3. Distribution of respiratory system compliances considering airway opening pressure normalized to predicted body weight (CRS-AOP/PBW). A. Patients categorized according to the presence or not of obesity and acute respiratory distress syndrome (ARDS). p-value represents the overall comparison between the four groups of patients. *, p < 0.05; 0**, p < 0.01; ***, p < 0.001 (pairwise comparisons with Bonferroni correction). B Patients categorized according to the presence or not of obesity and the presence or not of ARDS. p-values represent the comparisons between obese and non-obese patients and between ARDS and non-ARDS patients. Boxplots display medians, 10th, 25th, 75th, and 90th percentiles. Fig. S4. Correlations between respiratory [file 13054_2023_4623_MOESM1_ESM.docx]

**Additional data**

**Advanced respiratory mechanics assessment in mechanically ventilated obese and non-obese patients with or without acute respiratory distress syndrome**

François Beloncle, Jean-Christophe Richard, Hamid Merdji, Christophe Desprez, Bertrand Pavlovsky, Elise Yvin, Lise Piquilloud, Pierre-Yves Olivier, Dara Chean, Antoine Studer, Antonin Courtais, Maëva Campfort, Hassene Rahmani, Arnaud Lesimple, Ferhat Meziani and Alain Mercat.

**Table S1. Main characteristics of the patients according to the presence or not of ARDS and obesity.**

|  | **All patients**  **n = 149** | **Non-obese**  **n = 97** | **Obese**  **n = 52** | ***Obese vs non-obese p-*value** | **Non-ARDS**  **n = 59** | **ARDS**  **n = 90** | ***ARDS vs non-ARDS p-value***  ***p-value*** | |
| --- | --- | --- | --- | --- | --- | --- | --- | --- |
| **Age - years** | 65 [56-77] | 66 [55 - 77] | 65 [61 - 76] | 0.83 | 67 [56-78] | 64 [54-74] | 0.247 | |
| **Male sex - n** | 91 [61.1) | 57 (58.8) | 34 (65.4) | 0.43 | 37 (62.7) | 54 (60) | 0.864 | |
| **Height - cm** | 168 [160-174] | 168 [160 - 174] | 170 [162-174] | 0.32 | 168 [159-174] | 168 [161-175] | 0.446 |  |
| **BMI – kg.m^-2^** | 26 [23-32] | 24 [22-26] | 34 [31 - 39] | <0.001 | 25 [21-31] | 27 [24-33] | 0.100 | |
| **Abdominal perimeter** | 93 [104-115] | 96 [90 - 104] | 117 [114 - 131] | <0.001 | 98 [88-110] | 107 [96-117] | 0.064 | |
| **SOFA at enrollment** | 8 [6 -11] | 8 [6 - 11] | 8 [5 - 10] | 0.39 | 7 [6-9] | 9 [5-11] | 0.088 | |
| **Non-pulmonary SOFA at enrollment** | 5 [3.5-8] | 6 [4 – 8] | 5 [3 – 7] | 0.17 | 6 [2-8] | 5 [4-7] | 0.551 | |
| **SAPS II at enrollment** | 50 [39-63] | 52 [40 – 63] | 48 [37 – 60] | 0.24 | 50 [39-63] | 51 [36-61] | 0.668 | |
| **Chronic pulmonary disease** | 36 (24.2) | 15 (15.5) | 21 (40.4) | 0.003 | 14 (23.7) | 22 (24.4) | 0.982 | |
| **Immunodepression** | 18 (12.1) | 15 (15.5) | 3 (5.8) | 0.084 | 6 (10.2) | 12 (13.3) | 0.835 | |
| **Cause of ICU admission** |  |  |  |  |  |  |  | |
| **Pneumonia** | 57 (38.3) | 40 (41.2) | 17 (32.7) | 0.306 | 3 (5.1) | 54 (60) | <0.001 | |
| **Hydrostratic pulmonary edema** | 14 (9.4) | 6 (6.2) | 8 (15.4) | 0.067 | 14 (23.7) | 0 (0) | <0.001 | |
| **Non-pulmonary Sepsis** | 21 (14.1) | 13 (13.4) | 8 (15.4) | 0.740 | 6 (10.2) | 15 (16.7) | 0.265 | |
| **Acute exacerbation of obstructive pulmonary disease** | 3 (2) | 3 (3.1) | 0 (0) | 0.200 | 3 (5.1) | 0 (0) | 0.031 | |
| **Neurologic conditions / coma** | 28 (18.8) | 20 (20.6) | 8 (15.4) | 0.436 | 23 (39.0) | 5 (5.6) | <0.001 | |
| **Metabolic disorder** | 3 (2) | 1 (1) | 2 (3.8) | 0.244 | 2 (3.4) | 1 (1.1) | 0.333 | |
| **Other** | 23 (15.4) | 14 (14.4) | 9 (17.3) | 0.643 | 8 (13.6) | 15 (16.7) | 0.610 | |
| **Survival at day 60** | 101 (67.8) | 64 (66.0) | 37 (71.2) | 0.52 | 46 (78) | 55 (61.1) | 0.031 | |
| **Number of ventilator-free days at day 28 - days** | 13 [0 - 23] | 13 [0 - 24] | 13 [0 - 22] | 0.850 | 22 [0 - 26] | 5 [0 - 18] | <0.001 | |

*ARDS, Acute Respiratory Distress Syndrome; BMI, Body Mass Index; SOFA, Sequential Organ Failure Assessment; SAPS II, Simplified Acute Physiology Score II.* Data are presented as median [interquartile range] or number (percentage).

**Table S2. Gas exchange and respiratory mechanics of the patients according to the presence or not of ARDS and obesity.**

|  | **All patients**  **n = 149** | **Non-obese**  **n = 97** | **Obese**  **n = 52** | ***Obese vs non-obese***  ***p*-value** | **Non-ARDS**  **n =59** | **ARDS**  **n =90** | ***ARDS vs non-ARDS***  ***p-value*** |
| --- | --- | --- | --- | --- | --- | --- | --- |
| **RR - min^-1^** | 25 [20-30] | 25 [20 - 30] | 26 [22 - 30] | 0.077 | 22 [19-27] | 27 [25 -30] | <0.001 |
| **VE - L.min^-1^** | 9.6 [7.7-11.3] | 9.4 [7.6 - 11] | 9.9 [8.6 - 11.6] | 0.092 | 8.5 [6.8-10.2] | 10.2 [8.6-11.6] | <0.001 |
| **FiO_2_ - %** | 50 [30-70] | 40 [30 - 70] | 58 [40 - 70] | 0.049 | 35 [25-50] | 60 [40-70] | <0.001 |
| **PaO_2_ - mmHg** | 79 [69-96] | 78 [66 - 99] | 80 [70 - 92] | 0.82 | 82 [69-120] | 76 [67-89] | 0.021 |
| **PaO_2_ / FiO_2_ - mmHg** | 183 [120-255] | 190 [120 - 287] | 165 [121 - 224] | 0.052 | 270 [180-348] | 142 [104-198] | <0.001 |
| **PaCO_2_ - mmHg** | 40 [35-47] | 38 [34 - 46] | 42 [38 - 47] | 0.095 | 38 [33-45] | 42 [36-48] | 0.006 |
| **Ventilatory Ratio** | 1.3 [1.6 – 2] | 1.5 [1.2 - 1.9] | 1.8 [1.5 - 2.2] | 0.012 | 1.4 [1.1-1.6] | 1.8 [1.5-2.2] | <0.001 |
| **Patients with complete airway closure > 5 cmH_2_O - n** | 35 (23.5) | 14 (14.4) | 21 (40.4) | <0.001 | 8 (13.6) | 27 (30) | 0.029 |
| **AOP in patients with complete airway closure > 5 cmH_2_O - cmH_2_O** | 8.5 [7.5-11] | 9 [7 - 11.5] | 8.5 [8 - 10] | 0.91 | 8.5 [6.5 -12] | 8.5 [7.5-11] | 0.524 |
| **PEEP_tot_ - cmH_2_O** | 6 [5.5 -7.5] | 5.5 [5.5 - 7] | 7.0 [5.5 - 8.5] | <0.001 | 5 [5.5-6.5] | 5.5 [6.5-8] | 0.002 |
| **P_Plat_ - cmH_2_O** | 15 [13-18] | 14.5 [12.5 - 17] | 16 [15 - 19] | <0.001 | 14 [11-16] | 16 [14-19] | <0.001 |
| **C_RS_ - mL.cmH_2_O^-1^** | 44 [36-56] | 45 [36 - 59] | 42 [35 - 52] | 0.094 | 51 [39-66] | 41 [31-52] | 0.002 |
| **C_RS-AOP_ - mL.cmH_2_O^-1^** | 46 [36-57] | 47 [36 - 61] | 42 [36 - 53] | 0.23 | 52 [40-67] | 42 [35-53] | <0.001 |
| **DP_RS_ - cmH_2_O** | 8.5 [7-11] | 8 [6.5 - 10] | 9 [8 - 11] | 0.014 | 7.5 [6-9.5] | 9 [7-11] | 0.002 |
| **DP_RS-AOP_ - cmH_2_O** | 8.5 [6.5-10] | 8 [6 - 10.0] | 8.5 [7.5 - 10.0] | 0.068 | 7.5 [6-9] | 9 [7-11] | <0.001 |
| **R_RS_ - cmH_2_O.L^-1^.s^-1^** | 17 [14-21] | 16.5 [13.5 - 21] | 18[14 - 21.5] | 0.30 | 16.5 [14.5-21] | 17.5 [13.5-22] | 0.539 |
| **ΔP1-P_Plat_ - cmH_2_O** | 2.1 [1.1-3.4] | 1.8 [1.1-3] | 2.4 [1.1-4] | 0.248 | 1.7 [1-2.8] | 2.5 [1.3-4.8] | 0.042 |
| **DP_L_ - cmH_2_O** | 5 [4-7.5] | 5 [3.5-7] | 6 [5-7.5] | 0.058 | 4.5 [3-6.5] | 5.5 [4.5-8] | 0.018 |
| **P_Plat Lung_ - cmH_2_O** | 9.5 [7.5-12.5] | 9 [7 - 12] | 11 [9 - 12.5] | 0.021 | 8.5 [6.5-11] | 10 [8-12.5] | 0.010 |
| **Lung Stress - cmH_2_O** | 10 [7.5 - 14.5] | 9.5 [6.5 - 13] | 13.5 [9.5 - 16.5] | 0.006 | 9 [5 – 14.5] | 10.5 [8 - 15] | 0.030 |

*RR, Respiratory Rate; VE, minute ventilation; FiO_2_, fraction of inspired oxygen; PaO_2_, partial pressure of arterial oxygen; PaCO_2_, partial pressure of arterial carbon dioxide; AOP, Airway Opening Pressure; PEEP_tot_, total Positive End-Expiratory Pressure; P_Plat_, plateau Pressure; C_RS,_ Respiratory System Compliance; C_RS-AOP_, C_RS_ using AOP instead of PEEP_tot_ in the calculation; DP_RS,_ Respiratory System Driving Pressure;*

*DP_RS-AOP_, DP_RS_ using AOP instead of PEEP_tot_ in the calculation; R_RS_, Respiratory System Resistance; ΔP1-P_Plat_, difference between P1 and P_Plat_ with P1 defined as airway pressure at first zero flow; DP_L_, Lung Driving Pressure; P_Plat Lung_, plateau Pressure of the lung. Lung stress was defined as the difference between P_Plat Lung_ and the expiratory transpulmonary pressure.* Data are presented as median [interquartile range] or number (percentage).

**Table S3. Main characteristics of the patients according to the ratio of lung to respiratory system elastance (E_L_/E_RS_).**

|  | **Low E_L_/E_RS_**  **(n=31)** | **Medium E_L_/E_RS_**  **(n=62)** | **High E_L_/E_RS_**  **(n=31)** | **p-value** |
| --- | --- | --- | --- | --- |
| **Age - years** | 69 [59-77] | 67.5 [55-77] | 64 [47-71] | 0.208 |
| **Male sex - n** | 21 (67.7) | 37 (59.7) | 14 (45.2) | 0.187 |
| **Height - cm** | 170 [161-176] | 168 [160-174] | 163 [159-171] | 0.138 |
| **BMI – kg.m^-2^** | 24.3 [22-27.7] | 26.4 [22.8-32.5] | 26.8 [23.9-32.4] | 0.262 |
| **Obesity** | 5 (16.1) | 22 (35.5) | 11 (35.5) | 0.131 |
| **ARDS** | 18 (58.1) | 40 (64.5) | 21 (65.6) | 0.720 |
| **SOFA at enrollment** | 9 [6-12] | 8 [7-10] | 8 [5-11] | 0.678 |
| **Non-pulmonary SOFA at enrollment** | 6 [4-8] | 5.5 [4-7] | 4 [2-8] | 0.560 |
| **SAPS II at enrollment** | 51 [38-61] | 52 [40-65] | 47 [36-66] | 0.713 |
| **Chronic pulmonary disease** | 10 (32.3) | 11 (17.7) | 8 (25.8) | 0.217 |
| **Immunodepression** | 0 (0) | 10 (16.1) | 5 (16.1) | 0.073 |
| **Cause of ICU admission** |  |  |  |  |
| **Pneumonia** | 8 (25.8) | 23 (37.1) | 12 (38.7) | 0.480 |
| **Hydrostratic pulmonary edema** | 0 (0) | 9 (14.5) | 5 (16.1) | 0.070 |
| **Non-pulmonary Sepsis** | 4 (12.9) | 8 (12.9) | 6 (19.4) | 0.677 |
| **Acute exacerbation of obstructive pulmonary disease** | 4 (12.9) | 2 (3.2) | 3 (9.7) | 0.376 |
| **Neurologic conditions / coma** | 7 (22.6) | 7 (11.3) | 4 (12.9) | 0.331 |
| **Metabolic disorder** | 0 (0) | 2 (3.2) | 1 (3.2) | 0.599 |
| **Other** | 8 (25.8) | 11 (17.8) | 0 (0) | 0.014 |
| **Survival at day 60** | 9 (29) | 20 (32.2) | 13 (41.9) | 0.526 |
| **Number of ventilator-free days at day 28 - days** | 17 [0 - 25] | 14 [0 - 22] | 6 [0 - 18] | 0.427 |

*ARDS, Acute Respiratory Distress Syndrome; BMI, Body Mass Index; SOFA, Sequential Organ Failure Assessment; SAPS II, Simplified Acute Physiology Score II;* Data are presented as median [interquartile range] or number (percentage). P-values refer to the comparisons between the three groups of patients.

|  | **Low E_L_/E_RS_**  **(n=31)** | **Medium E_L_/E_RS_**  **(n=62)** | **High E_L_/E_RS_**  **(n=31)** | **p-value** |
| --- | --- | --- | --- | --- |
| **RR - min^-1^** | 22 [20-28] | 26 [22-30] | 26 [24-30] | 0.070 |
| **VE - L.min^-1^** | 8.7[7.6-11.4] | 10 [8-11.3] | 9.4 [8-10.9] | 0.371 |
| **FiO_2_ - %** | 40 [30-60] | 50 [30-60] | 60 [40-80] | 0.002 |
| **PaO_2_ - mmHg** | 79 [67-94] | 73 [64-90] | 80 [71-97] | 0.453 |
| **PaO_2_ / FiO_2_ - mmHg** | 222 [138-303] | 183 [116-246] | 138 [116-200] | 0.027 |
| **PaCO_2_ - mmHg** | 43 [36-52] | 38 [34-44] | 42 [35-48] | 0.021 |
| **Patients with complete airway closure > 5 cmH_2_O - n** | 10 (32.3) | 13 (20.9) | 7 (22.6) | 0.496 |
| **AOP in patients with complete airway closure > 5 cmH_2_O - cmH_2_O** | 8 [7-8.5] | 9 [7.5-11.2] | 10 [8.5-12.4] | 0.116 |
| **EELV/PBW – mL.kg^-1^** | 25 [19.7-36.9] | 21.6 [17.8-25.9] | 17 [12.9-21.5] | <0.001 |
| **PEEP_tot_ - cmH_2_O** | 6 [5.5-8] | 6 [5.5-7.5] | 6 [5.5-7.5] | 0.850 |
| **P_Plat_ - cmH_2_O** | 14 [11.5-16] | 14.5 [13-18] | 17 [15.5-20] | <0.001 |
| **C_RS_ - mL.cmH_2_O^-1^** | 53 [43-74] | 43 [37-56] | 35 [26-44] | <0.001 |
| **C_RS_ /PBW - mL.cmH_2_O^-1^.kg^-1^** | 0.92 [0.67-1.18] | 0.75 [0.6-0.89] | 0.62 [0.43-0.67] | <0.001 |
| **C_RS-AOP_/PBW - mL.cmH_2_O^-1^.kg^-1^** | 0.92 [0.67-1.18] | 0.77 [0.61-0.90] | 0.62 [0.43-0.73] | <0.001 |
| **C_L_ - mL.cmH_2_O^-1^** | 113 [94 - 181] | 68 [54-93] | 40 [29-50] | <0.001 |
| **C_L_ /PBW - mL.cmH_2_O^-1^.kg^-1^** | 1.9 [1.40-2.61] | 1.15 [0.94-1.43] | 0.75 [0.50-0.87] | <0.001 |
| **C_CW_ - mL.cmH_2_O^-1^** | 100 [81-131] | 125 [106-152] | 198 [145-286] | <0.001 |
| **DP_RS_ - cmH_2_O** | 6.5 [5.5-9] | 8.5 [6.5-10] | 10 [9-15] | <0.001 |
| **DP_RS-AOP_ - cmH_2_O** | 6.5 [5.5-9] | 8 [6.5-9.5] | 10 [8.5-15] | <0.001 |
| **R_RS_ - cmH_2_O.L^-1^.s^-1^** | 19 [14-22.5] | 17 [13.5-20] | 17.5 [14.5-21] | 0.362 |
| **ΔP1-P_Plat_ - cmH_2_O** | 2 [0.9 - 4.4] | 1.8 [1.1 - 3.2] | 2.5 [1.1 - 3.5] | 0.824 |
| **P_eso expi_ - cmH_2_O** | 6 [4-10] | 7.5 [5.5-9.5] | 8.5 [5-10.5] | 0.264 |
| **P_L expi_ - cmH_2_O** | 0.5 [-1-2] | -1 [-3.5-1] | -1 [-4-1.5] | 0.107 |
| **P_abdo_ - cmH_2_O** | 11 [5-13] | 8.5 [5-11] | 9 [6-16] | 0.780 |
| **P_Plat Lung_ - cmH_2_O** | 6.5 [5-7.5] | 9.5 [8-11.5] | 13.5 [12-18] | <0.001 |
| **DP_L_ - cmH_2_O** | 3 [2.5-4.5] | 5 [4.5-6.5] | 8.5 [7-12] | <0.001 |
| **Lung Stress - cmH_2_O** | 5.5 [ 3.5 - 9] | 10 [8 – 13.5] | 16.5 [12.5 – 21.5] | <0.001 |

**Table S4. Gas exchange and respiratory mechanics of the patients according to the ratio of lung to respiratory system elastance (E_L_/E_RS_).**

*RR, Respiratory Rate; VE, minute ventilation; FiO_2_, fraction of inspired oxygen; PaO_2_, partial pressure of arterial oxygen; PaCO_2_, partial pressure of arterial carbon dioxide; AOP, Airway Opening Pressure; EELV, End-Expiratory Lung volume; PBW, Predicted Body Weight; PEEP_tot_, total Positive End-Expiratory Pressure; P_Plat_, plateau Pressure; C_RS,_ Respiratory System Compliance; C_RS-AOP_, C_RS_ using AOP instead of PEEP_tot_ in the calculation; C_L_, Lung Compliance; C_CW_, Chest Wall Compliance; DP_RS,_ Respiratory System Driving Pressure; DP_RS-AOP_, DP_RS_ using AOP instead of PEEP_tot_ in the calculation; R_RS_, Respiratory System Resistance; ΔP1-P_Plat_, difference between P1 and P_Plat_ with P1 defined as airway pressure at first zero flow; P_eso expi_, Expiratory Esophageal Pressure; P_L expi_, Expiratory transpulmonary Pressure; P_abdo_, Abdominal Pressure; DP_L_, Lung Driving Pressure; P_Plat Lung_, plateau Pressure of the Lung; Lung stress was defined as the difference between P_Plat Lung_ and P_L expi_.* Data are presented as median [interquartile range] or number (percentage). P-values refer to the comparisons between the three groups of patients.

**Fig. S1.** Correlations between the ratio of partial pressure of arterial oxygen over fraction of inspired oxygen (PaO_2_ / FiO_2,_ **A**) and Ventilatory Ratio (VR, **B**), and body mass index (BMI) in patients with or without acute respiratory distress syndrome (ARDS).

**Fig. S2.** Distribution of respiratory system compliance (C_RS_/PBW, **A**), lung compliances (C_L_/PBW, **B**) and end-expiratory lung volume (EELV/PBW, **C**) normalized to predicted body weight in patients categorized according to the presence or not of obesity and the presence or not of acute respiratory distress syndrome (ARDS). Boxplots display medians, 10^th^, 25^th^, 75^th^, and 90^th^ percentiles. P-values represent the comparisons between obese and non-obese patients and between ARDS and non-ARDS patients.

**Fig. S3.** Distribution of respiratory system compliances considering airway opening pressure normalized to predicted body weight (C_RS-AOP_/PBW).

**A.** Patients categorized according to the presence or not of obesity and acute respiratory distress syndrome (ARDS). P-value represents the overall comparison between the four groups of patients. *, p<0.05; **, p<0.01; ***, p<0.001 (pairwise comparisons with Bonferroni correction).

**B.** Patients categorized according to the presence or not of obesity and the presence or not of ARDS. P-values represent the comparisons between obese and non-obese patients and between ARDS and non-ARDS patients.

Boxplots display medians, 10^th^, 25^th^, 75^th^, and 90^th^ percentiles.

**Fig. S4.** Correlations between respiratory system compliance (C_RS_/PBW, **A**) and end-expiratory lung volume normalized to predicted body weight (EELV/PBW, **B**) and body mass index (BMI) in patients with or without acute respiratory distress syndrome (ARDS).

**Fig. S5.** Correlations between respiratory system compliance (C_RS_) and end-expiratory lung volume (EELV) at positive end-expiratory pressure of 5 cmH_2_O in obese and non-obese patients with or without acute respiratory distress syndrome (ARDS).

**Fig. S6.** Distribution of chest wall compliance (C_CW_, **A**) and end-expiratory esophageal pressure (P_eso expi_, **B**) in patients categorized according to the presence or not of obesity and the presence or not of acute respiratory distress syndrome (ARDS). Boxplots display medians, 10^th^, 25^th^, 75^th^, and 90^th^ percentiles. P-values represent the comparisons between obese and non-obese patients and between ARDS and non-ARDS patients.

**Fig. S7.** Correlations between chest wall compliance (C_cw_, **A**) and expiratory esophageal pressure (P_eso expi_, **B**) and body mass index (BMI) in patients with or without acute respiratory distress syndrome (ARDS).

**Fig. S8.** Distribution of lung to respiratory system elastance ratio (E_L_/E_RS_).

**A.** Patients categorized according to the presence or not of obesity and acute respiratory distress syndrome (ARDS). P-value represents the overall comparison between the four groups of patients.

**B.** Patients categorized according to the presence or not of obesity and the presence or not of ARDS. P-values represent the comparisons between obese and non-obese patients and between ARDS and non-ARDS patients.

Boxplots display medians, 10^th^, 25^th^, 75^th^, and 90^th^ percentiles.
